# Delay-Adjusted Age-Specific COVID-19 Case Fatality Rates in a High Testing Setting: South Korea, February 2020 to February 2021

**DOI:** 10.3390/ijerph18105053

**Published:** 2021-05-11

**Authors:** Eunha Shim

**Affiliations:** Department of Mathematics, Soongsil University, Seoul 06978, Korea; alicia@ssu.ac.kr; Tel.: +82-(02)-820-0416

**Keywords:** SARS-CoV-2, COVID-19, Republic of Korea, age-specific death rate, case fatality rate

## Abstract

In South Korea, a country with a high coronavirus disease 19 (COVID-19) testing rate, a total of 87,324 COVID-19 cases, including 1562 deaths, have been recorded as of 23 February 2021. This study assessed the delay-adjusted COVID-19 case fatality risk (CFR), including data from the second and third waves. A statistical method was applied to the data from 20 February 2021 through 23 February 2021 to minimize bias in the crude CFR, accounting for the survival interval as the lag time between disease onset and death. The resulting overall delay-adjusted CFR was 1.97% (95% credible interval: 1.94–2.00%). The delay-adjusted CFR was highest among adults aged ≥80 years and 70–79 years (22.88% and 7.09%, respectively). The cumulative incidence rate was highest among individuals aged ≥80 years and 60–69 years. The cumulative mortality rate was highest among individuals aged ≥80 years and 70–79 years (47 and 12 per million, respectively). In South Korea, older adults are being disproportionately affected by COVID-19 with a high death rate, although the incidence rate among younger individuals is relatively high. Interventions to prevent COVID-19 should target older adults to minimize the number of deaths.

## 1. Introduction

As of 23 February 2021, more than 110 million cases of coronavirus disease-19 (COVID-19) and approximately 2.5 million deaths had been reported worldwide [1]. The ongoing COVID-19 pandemic has imposed a substantial burden on health systems and economies, overwhelming many health systems worldwide [2]. Estimation of case fatality rates (CFRs) is a key epidemiological metric used to assess the severity of an epidemic, allowing public health officials to determine the type and intensity of intervention strategies required to mitigate disease transmission [3].

As of 23 February 2021, South Korea, a country of 52 million people, had reported 87,324 cases of COVID-19 and 1562 deaths [4]. The majority (62%) of COVID-19 cases in South Korea have been reported in metropolitan areas, namely Seoul, Incheon and Gyeonggi Province [4]. The rapid implementation of extensive testing in South Korea is considered to have been successful in mitigating the spread of the disease, without requiring the need for drastic measures such as a complete city lockdown [5]. Despite these successful measures, a second wave of the pandemic started in South Korea in August 2020, with multiple clusters of infections linked to Protestant churches in the metropolitan Seoul area [4]. In the third week of November 2020, the greater Seoul area was considered to have entered a third wave of COVID-19, with the daily caseload remaining over 100 since November 8. There were 313 cases confirmed on November 18, exceeding 300 for the first time since late August. In light of rapid surges in COVID-19 cases, the Korean government raised the social distancing guidance level in Seoul to level 2—the third highest in a five-tier social distancing scheme—on 24 November 2020, which included recommendations such as one-third of the workforce working from home, staggering work and lunch hours, mask wearing in all indoor spaces, and suspending all nonessential business trips [4]. Following this, the social distancing level in other areas outside Seoul was raised to 1.5 on 29 November [4]. On 8 December, the social distancing level was raised to level 2.5, the second highest in the country’s five-tier scheme, in metropolitan areas, and to level 2 in other areas [4]. As of 23 February 2021, South Korea was still experiencing a third wave of COVID-19 [6].

Despite the implementation of these nonpharmaceutical interventions in South Korea, the number of cases and deaths have continued to rise. Severity estimates for COVID-19 are essential to assess the potential impact of the ongoing pandemic. The crude CFR is defined as the cumulative number of deaths divided by the cumulative number of cases. As of 23 February 2021, the crude CFR in South Korea was estimated to be 1.79%, which was lower than the global average crude CFR of 2.22% [1]. However, the crude CFR does not accurately capture the increase in the number of fatal cases because of the delay between diagnosis and death; so called right-censoring. Therefore, it may underestimate the severity of the pandemic. However, the crude CFR may also overestimate the probability of death because the denominator is calculated based only on the number of confirmed cases. This is known as ascertainment bias, which describes the phenomenon of a surveillance system being much more likely to detect and diagnose severe and fatal cases than mild ones. The spectrum of symptomatic infections ranges from mild symptoms to critical conditions, which are characterized as respiratory failure, shock, and multiorgan dysfunction, and can result in death. Asymptomatic infections of COVID-19 have also been well documented, with 33% to 73% of all infected individuals being asymptomatic [7,8,9]. In South Korea, however, the percentage of COVID-19 positive tests has been reported to be relatively low (1.4%) and ranked the eleventh lowest positivity rate worldwide, which reflects the proactive implementation of widespread testing for COVID-19 and contact tracing for positive cases [6,10]. Furthermore, widespread testing undertaken by the Korea Disease Control and Prevention Agency (KDCA) has been considered to have effectively identified asymptomatic individuals, and case ascertainment in South Korea has been considered more complete than in many other settings, thus reducing ascertainment bias toward severe cases [10,11].

To date, most published COVID-19 CFR estimates have not provided age-stratified estimates and have not reported on settings where case ascertainment is likely to be relatively complete. However, it is known that middle-aged and older adults have a higher incidence of severe acute respiratory syndrome coronavirus 2 (SARS-CoV-2) infection than younger individuals, older adults are more likely to develop severe COVID-19 [12], and COVID-19 mortality increases with age [7,13,14]. The Chinese Center for Disease Control and Prevention has reported CFRs of 8% and 15% among those aged 70–79 years and ≥80 years, respectively, in contrast to an all-age CFR of 2.3% [7]. Similarly, in the United Kingdom, the CFR among individuals aged ≥80 years is 20-fold higher than that among individuals aged 50–59 years [7]. Thus, it is essential to assess the age-specific CFR to gauge the severity of the SARS-CoV-2 pandemic.

The global CFR of COVID-19 was reported to be 3% in initial studies [15], whereas country-specific crude CFRs have ranged from the lowest rates in Germany (0.7%) and South Korea (2.4%) to the highest rates in Canada (4.9%), the United States (5.4%), Spain (6.0%), the Netherlands (7.4%) and Italy (9.3%) [16,17,18]. To reduce the bias of the crude CFR, several CFR estimators have been proposed. For instance, prior studies have proposed adjusting the number of confirmed cases in the denominator by multiplying a factor [19,20,21,22,23]. In a prior study concerning the CFR of COVID-19 in Canada, we showed that the estimated crude CFR would be 2.87% on 15 December 2020, whereas the adjusted CFR was estimated at 3.36% (credible interval [CrI] 3.29–3.43%) [24]. A previous study by Shim et al. on the risk of COVID-19-related death in South Korea estimated delay-adjusted CFRs but did not report age-stratified estimates [18]. Another study by Newall et al. [11] estimated age-specific CFRs for South Korea but only included incidence and mortality data up to 12 June 2020, which comprised mainly data from the first wave, during which the reported crude CFR (2.31%) was higher than that reported in this study. In addition, based on data up to June 2020, the highest incidence occurred among younger adults aged 20–29 years, whereas the cumulative age distribution of cases as of 23 February 2021 was closer to the South Korean population distribution. Hence, it is important to reassess the age-specific CFR based on updated data, including data from the second and third waves of the pandemic in South Korea.

This study aimed to estimate the risk of death among confirmed cases, taking into account ascertainment bias and right-censoring using established methods [20,25]. Given the importance of delay-adjusted CFR estimates according to age, this study aimed to provide real-time estimates of adjusted age-specific CFR during the COVID-19 pandemic, and to identify the most vulnerable segments of the population in South Korea, through February 2021. The age-specific delay-adjusted CFR estimates in South Korea could be useful for estimating the impact of the COVID-19 pandemic in South Korea where large-scale testing has been implemented as a core disease control intervention.

This paper is organized as follows. In Section 2, the data sources and the method to calculate delay-adjusted COVID-19 CFRs are presented. In Section 3, the results are presented, which include epidemiological characteristics of COVID-19 in South Korea and crude and delay-adjusted CFRs. Section 4 and Section 5 focus on discussion and conclusions, respectively.

## 2. Materials and Methods

### 2.1. Data Sources

Daily cumulative numbers of COVID-19 cases and deaths stratified according to age group were obtained from daily reports published by the KDCA [4]. The analysis used data reported from 20 February 2021 through 23 February 2021, from which the number of stratified polymerase chain reaction (PCR)-confirmed cases and deaths according to age groups was extracted, forming a time series. A confirmed diagnosis of COVID-19 was based on the detection of SARS-CoV-2 RNA using reverse-transcription PCR (RT-PCR). According to the KDCA’s Center for Laboratory Control of Infectious Diseases, the criterion for negative RT-PCR for SARS-CoV-2 is a cycle threshold (Ct) value of ≥37, whereas the positive upper limit of the Ct value is 35 [26]. The daily updates on COVID-19 cases were reported in age groups at 10-year intervals. Given there was one COVID-19 death recorded in those aged <30 years in South Korea during the period of analysis, the age groupings used for the analysis were 0–29, 30–39, 40–49, 50–59, 60–69, 70–79 and 80+ years. Ministry of Health and Welfare (MOHW) announcements were used to confirm the validity of the time series data.

### 2.2. Statistical Analysis

The denominator of the crude CFR formula includes infected people whose outcomes are not yet known, including individuals who have not yet died from the disease but will do so in the future. Thus, the delay between infection and death results in bias when calculating the CFR, known as right censoring [27]. Statistical methods developed by Nishiura et al. [20] were used to estimate the delay-adjusted age-specific CFRs. Specifically, the factor of adjustment,$\;u_{t}$, was defined as:

$$u_{t}=\frac{\sum_{i=0}^{t}\sum_{j=0}^{\infty }c_{i-j}f_{j}}{\sum_{i=0}^{t}c_{i}}$$

where $c_{t}$ is the number of new confirmed cases on day *t*, and $f_{t}$ is the conditional probability density function of the time from onset to death [20]. Under the assumption of an exponential increase of onsets with growth rate *r*, the expectation of incidence *E*(*c*(*t*)) can be written as:

$$E\left(c\left(t\right)\right)=c_{0}e^{rt}$$

where $c_{0}$ is a constant and *r* is an intrinsic growth rate.

Using the equation above, the factor of underestimation can be rewritten as:

$$u=\int_{0}^{\infty }\exp\left(-rs\right)f\left(s\right)ds.$$

If *f*(*s*) is the density of a gamma distribution, which is consistent with prior findings [28,29], with mean *T* and coefficient of variation *v*, an adjustment factor, *u*(*t*), can be used and defined as:

$$u\left(t\right)={\left(1+rTv^{2}\right)}^{-1/v^{2}}.$$

The maximum likelihood method was used to estimate the growth rate of the epidemic during COVID-19 by age group. To calculate the adjustment factor, *u_a_*, incidence data and the distribution of time from disease onset to death were used. Specifically, we considered the distribution of the time from onset to death, *F*(*s*), where *F*(*s*) was the density of a gamma distribution with a mean *T* (11.5 days) and a standard deviation (*v^2^*) of 6.6 days [4,11,20].

Using the adjustment factor, the following equation was employed to create an unbiased estimator of the CFR, *p_t_*:

$$p_{t}=\frac{b_{t}}{u_{t}}$$

where *b_t_* is a crude biased estimated CFR calculated at time *t* [20]. To account for the uncertainty in time from disease onset to death, and to generate 95% confidence intervals (CIs) in the CFR estimates, the distribution *F*(*s*) was varied by sampling the mean from a normal distribution with a mean of 11.5 days and a standard deviation of one day. The moment-generating function was then used to determine the adjusted CFR on each calendar day by running Monte Carlo simulations with 1000 independent replications [20].

## 3. Results

### 3.1. Epidemiological Characteristics of COVID-19 in South Korea

**Figure 1:**
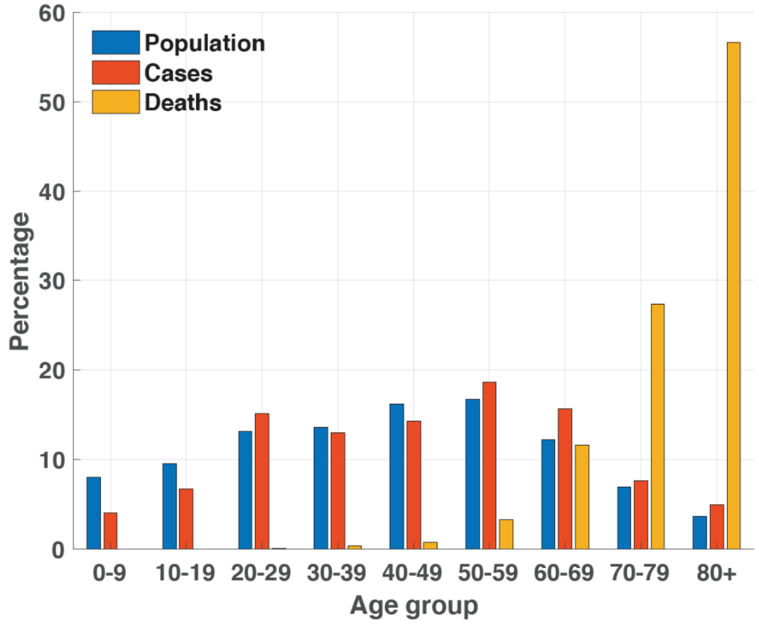
Distribution of the population, COVID-19 cases, and COVID-19 deaths according to age in South Korea, as of 23 February 2021.

Among a total of 87,681 COVID-19 cases reported by the KDCA up to 23 February 2021, most reported cases involved persons aged 50–59 years (18.6%), followed by those aged 60–69 years (15.7%) and 20–29 years (15.1%) (Figure 1) [4]. In contrast, the reported deaths due to COVID-19 increased substantially with age, with 20.7% of a total 1573 deaths occurring among those aged ≥80 years, followed by those aged 70–79 years (6.4%) and 60–69 years (1.3%; Figure 1). There were no COVID-19 deaths recorded among individuals aged <20 years as of 23 February 2021.

**Figure 2:**
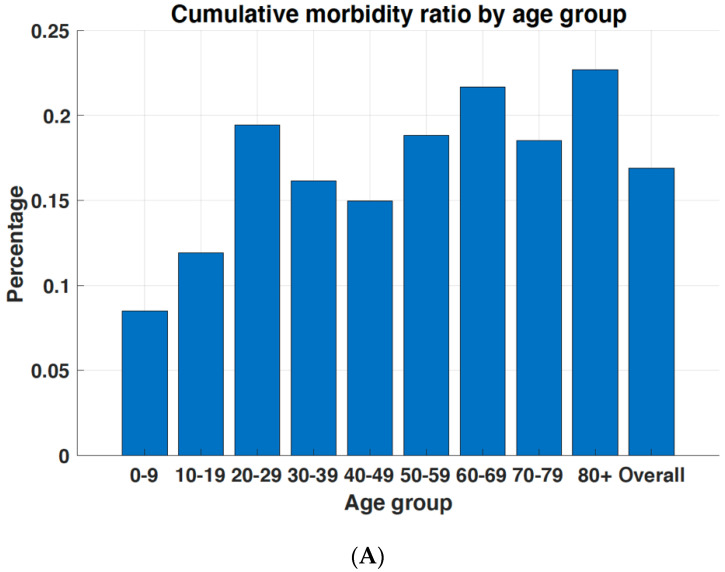

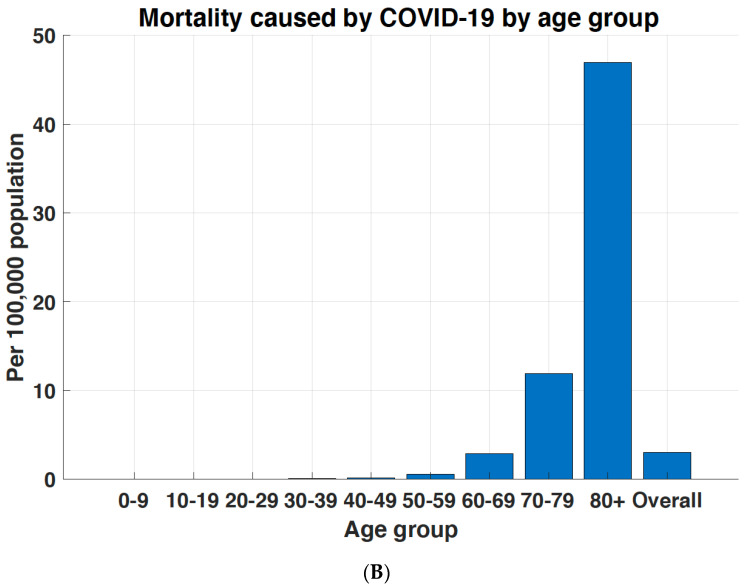
Mortality rate due to COVID-19 according to age group in South Korea as of 23 February 2021. (**A**) Cumulative COVID-19 incidence rate according to age group; (**B**) COVID-19 mortality rate according to age group.

Figure 2A shows the cumulative incidence rate according to age group. The cumulative incidence rate across all ages was 0.17%, with the highest rate (0.23%) among individuals aged ≥80 years, followed by those aged 60–69 years (0.22%). The mortality rate per 100,000 population directly in relation to COVID-19 according to age group is shown in Figure 2B. Individuals aged ≥80 years were most affected. Specifically, the mortality rate per 100,000 was 46.9 among individuals aged ≥80 years, and 11.9 among those aged 70–79 years, compared with 3.0 for all ages.

### 3.2. Evolution of Cases and Deaths According to Age Group

**Figure 3:**
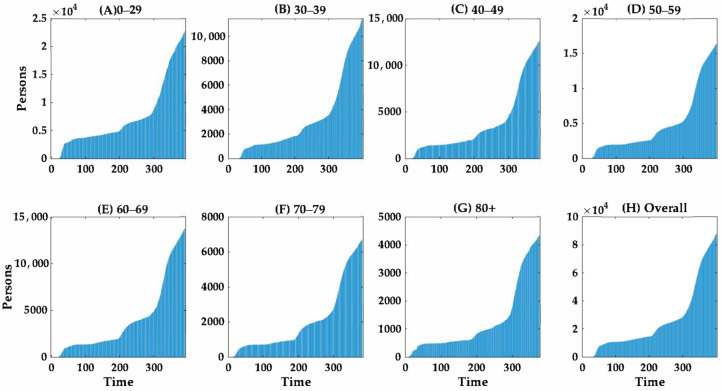
Temporal distribution of cumulative COVID-19 cases according to age group in South Korea from 20 February 2021 to 23 February 2021. The cumulative number of COVID-19 cases are shown according to the following age groups: (**A**) 0–29 years, (**B**) 30–39 years, (**C**) 40–49 years, (**D**) 50–59 years, (**E**) 60–69 years, (**F**) 70–79 years, (**G**) ≥80 years and (**H**) all cases. Day 1 corresponds to 20 February 2021. The scale on the *Y*-axis differs according to age group.

**Figure 4:**
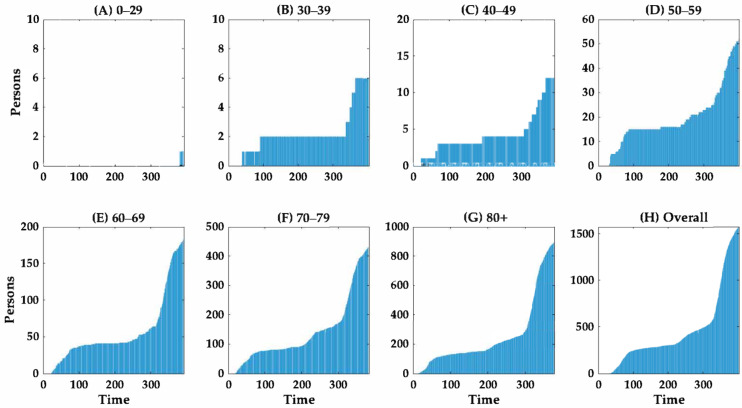
Temporal distribution of cumulative COVID-19 deaths according to age group in South Korea from 20 February 2021 to 23 February 2021. The cumulative number of COVID-19 deaths are shown according to the following age groups: (**A**) 0–29 years, (**B**) 30–39 years, (**C**) 40–49 years, (**D**) 50–59 years, (**E**) 60–69 years, (**F**) 70–79 years, (**G**) ≥80 years and (**H**) all COVID-19 deaths. Day 1 corresponds to 20 February 2021. The scale on the *Y*-axis differs according to age group.

The cumulative numbers of cases and deaths according to age group over time are shown in Figure 3 and Figure 4, respectively. The curves suggest that the cumulative number of cases of COVID-19 increased proportionally faster than the cumulative number of deaths. The growth curve for the cumulative number of cases across all age groups increased more rapidly after day 275 (20 November 2020), during the third wave, than prior to this date. Notably, the number of fatal cases increased dramatically with age (Figure 4).

### 3.3. Crude and Delay-Adjusted Risk of Death

**Figure 5:**
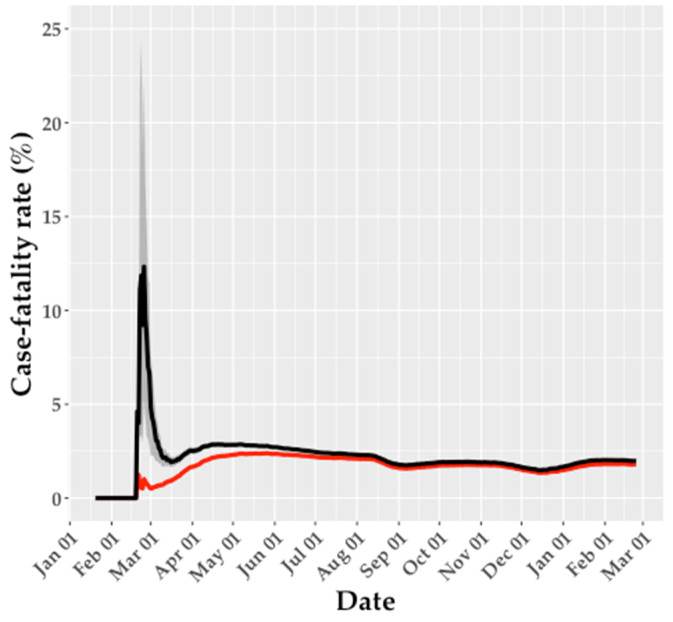
Crude case fatality rate (CFR; red line) and adjusted CFR estimates (black line) in South Korea up to 23 February 2021. The shaded area around the black line illustrates the 95% credible interval (CrI) for the adjusted CFR.

The crude and delay-adjusted CFRs were relatively similar, overall, although the delay-adjusted CFR was highly unstable early in the epidemic (Table 1 and Figure 5). The early rise in the adjusted CFR occurred because both the number of deaths and the number of cases reported increased at approximately the same time in late February 2020. During this period, the denominator, that is, the number of cases predicted to have a known outcome (survived or died), was relatively low, resulting in unstable estimates of the adjusted CFR. However, the delay-adjusted CFR stabilized over time because the outcomes of most cases, either recovery or death, were known by the endpoint of the analysis when enough time had elapsed from the peak in cases.

Both the crude and delay-adjusted CFR estimates increased substantially with age. Among all age groups, older adults were the most severely affected. The time-delay adjusted CFRs were 22.88% (95% CrI: 22.48–23.29%) for those aged ≥80 years and 7.09% (95% CrI: 7.00–7.21%) among those aged 70–79 years, compared to an overall CFR of 1.97% (95% CrI: 1.94–2.00%) for all ages combined.

## 4. Discussion

In this study, the time-delay adjusted CFR according to age group was estimated for the ongoing COVID-19 pandemic in South Korea. Consistent with previous studies that estimated the time-delay adjusted COVID-19 CFR according to age group [11,30,31], both the crude and the delay-adjusted CFRs for South Korea increased rapidly with age. Specifically, it was shown that the COVID-19 pandemic in South Korea has disproportionately affected adults aged ≥70 years, despite the relatively low overall adjusted CFR (1.97%) for all ages combined. This finding highlights the importance of determining age-specific CFRs, in addition to the overall CFR, when assessing the risk of mortality associated with COVID-19. Furthermore, this study found that both the delay-adjusted and crude CFRs showed substantial variations especially at the beginning of the pandemic in South Korea. Similar patterns have been observed in previous studies concerning delay-adjusted CFRs conducted in South Korea and other countries [11,25,31,32]. Thus, these results highlight that caution must be applied when interpreting CFRs, particularly earlier in the pandemic, even with adjustment for the delay between diagnosis and death. Moreover, there is likely to have been substantial variation in terms of delay in notification of confirmed cases and deaths throughout the pandemic, further complicating the interpretation of CFRs in the early phase, partially due to changes in surveillance and reporting practices. In the United Kingdom, the reporting delay is reported to have decreased over time [33].

The age structure of affected populations should be considered when comparing CFRs among countries. Therefore, age-specific estimates of the CFR would be very useful when comparing CFRs of countries with differing age structures. Age-specific CFRs suggest that countries with aging populations may experience higher COVID-19-related CFRs than countries with more youthful populations, as shown in Italy [14]. While the population in South Korea is aging at a rapid rate, the proportion of the population aged ≥65 years is 13.9%, which is relatively low in comparison to highly affected countries such as the U.S. (15.4%), Spain (19.4%), France (19.7%), Germany (21.5%), Bulgaria (20.8%) and Italy (23.0%) [34]. In China and Italy, COVID-19 cases have primarily involved older adult populations, whereas in South Korea a higher proportion of younger individuals (aged 20–29 years) have been affected than in other countries [35]. In Korea, the relatively low CFR is likely to reflect the age distribution of the cases, and could also have resulted from the larger proportion of cases being confirmed among younger individuals, leading to a larger denominator in relation to this low-risk population. Hence, the demographic distribution of cases in South Korea, good access to healthcare, and the population age structure, favor a lower overall CFR than in countries with a relatively older population [36].

This study has some limitations. First, while the successful control of the pandemic in South Korea suggests a relatively high level of case ascertainment, a seroprevalence survey conducted in Daegu found evidence of substantial numbers of undiagnosed cases in South Korea [37]. The CFR estimates are likely to be affected by under ascertainment, which might result in overestimation of the CFR if the surveillance system preferentially captured the most severe cases, as has been reported in some other countries [38,39,40]. Second, South Korean data include a proportion of asymptomatic cases but not all asymptomatic cases; thus, these estimates represent neither a complete estimate of the CFR among symptomatic cases of COVID-19 nor of the CFR involving all cases with SARS-CoV-2 infections. Third, the demographic distribution of cases has evolved over time throughout the pandemic, which affects the delay in reporting due to the age-related variation in susceptibility to symptomatic infection. Younger individuals who generally suffer milder (or asymptomatic) infection may have been under-represented as a result, and/or had a longer delay from symptom onset to death. The COVID-19 pandemic in South Korea moved from initial clusters related to a religious group in Daegu, predominantly affecting females aged 20–29 years, to involve more widely distributed cases among all age groups in the community. This shift might have affected the delay, but the distribution of delay was fixed in this analysis [41]. Furthermore, the publicly available data from the KDCA did not include daily data stratification according to age and sex for the entire period; thus, the estimates of CFR in this study were stratified only according to age and not according to sex. Although important differences in CFR according to age were identified in this study, future research should include other independent predictors of mortality risk for COVID-19, such as pre-existing conditions including hypertension, diabetes mellitus and cardiovascular disease, and risk factors such as an increased body mass index [42].

## 5. Conclusions

This study used real-time epidemiological data in South Korea, which is a setting with high rates of testing for COVID-19, and found that older adults, especially those aged ≥70 years, were disproportionately affected due to the COVID-19 pandemic, consistent with previous studies conducted in other regions. These results suggest that intervention strategies against COVID-19 should target older adults and should seek to improve adherence with preventive measures among this group to maximize the effect of interventions on minimizing the number of deaths.

## Figures and Tables

**Table 1:** Summary results of cumulative time-delay-adjusted COVID-19 case fatality rates according to age group in South Korea (23 February 2021).

Age Group (Years)	Latest Estimate (95% CrI)	Range of Median Estimates	Crude Case Fatality Rate
0–29	0.00% (0.00–0.01%)	0.00–0.01%	0.00%
30–39	0.06% (0.06–0.06%)	0.04–6.17%	0.05%
40–49	0.11% (0.10–0.11%)	0.09–27.17%	0.10%
50–59	0.35% (0.34–0.36%)	0.29–20.41%	0.32%
60–69	1.46% (1.44–1.49%)	1.14–37.35%	1.32%
70–79	7.09% (7.00–7.21%)	5.75–29.13%	6.41%
80+	22.88% (22.48–23.29%)	16.57–35.95%	20.61%
All ages	1.97% (1.94–2.00%)	1.49–12.33%	1.79%

CrI: credible interval.

## Data Availability

The daily number of confirmed cases and deaths associated with COVID-19 in South Korea was obtained from publicly available sources, available at https://www.cdc.go.kr (accessed on 15 April 2021).
